# Predictive short/long-term efficacy biomarkers and resistance mechanisms of CD19-directed CAR-T immunotherapy in relapsed/refractory B-cell lymphomas

**DOI:** 10.3389/fimmu.2023.1110028

**Published:** 2023-03-27

**Authors:** Hao Xu, Ningwen Li, Gaoxiang Wang, Yang Cao

**Affiliations:** ^1^Department of Hematology, Tongji Hospital, Tongji Medical College, Huazhong University of Science and Technology, Wuhan, Hubei, China; ^2^Immunotherapy Research Center for Hematologic Diseases of Hubei Province, Wuhan, Hubei, China

**Keywords:** biomarker, CAR-T therapy, B-cell lymphoma, refractory/relapse, efficacy, resistance

## Abstract

Genetically modified T-cell immunotherapies are revolutionizing the therapeutic options for hematological malignancies, especially those of B-cell origin. Impressive efficacies of CD19-directed chimeric antigen receptor (CAR)-T therapy have been reported in refractory/relapsed (R/R) B-cell non-Hodgkin lymphoma (NHL) patients who were resistant to current standard therapies, with a complete remission (CR) rate of approximately 50%. At the same time, problems of resistance and relapse following CAR-T therapy have drawn growing attention. Recently, great efforts have been made to determine various factors that are connected to the responses and outcomes following CAR-T therapy, which may not only allow us to recognize those with a higher likelihood of responding and who could benefit most from the therapy but also identify those with a high risk of resistance and relapse and to whom further appropriate treatment should be administered following CAR-T therapy. Thus, we concentrate on the biomarkers that can predict responses and outcomes after CD19-directed CAR-T immunotherapy. Furthermore, the mechanisms that may lead to treatment failure are also discussed in this review.

## Introduction

It has been more than ten years since the primary data of hematological malignancies that were resistant to standard therapies and successfully treated with chimeric antigen receptor (CAR)-T therapy were reported (1–3). CD19-directed CAR-T therapy achieved meaningful success in refractory/relapsed (R/R) chronic lymphocytic leukemia (CLL) patients, and the results warrant subsequent clinical trials that explore CAR-T therapy targeting different tumor antigens in various types of hematological malignancies. To date, several CAR-T products have been approved worldwide, which broaden the therapeutic options for R/R aggressive B-cell lymphoma, acute leukemia of B-cell origin and multiple myeloma.

CAR-T therapy targeting CD19 has been most widely studied. For R/R B-cell NHL, five CAR-T therapies, Tisagenlecleucel (tisa-cel, Kymriah), Brexucabtagene autoleucel (brexu-cel, Tecartus), Axicabtagene ciloleucel (axi-cel, Yescarta), Lisocabtagene maraleucel (liso-cel, Breyanzi) and relmacabtagene autoleucel (relma-cel, Carteyva), were FDA/NMPA approved. Several pivotal trials reported overall response rates (ORRs) between 52% and 82% (4–6). The long-term follow-up data revealed that the OS rates at 12 months were 49% to 59%, with progression-free survival (PFS) rates of 44% to 65% (4–6). Apart from the promising results, we should note the limitations that among patients who initially achieved response, the cancers of 21% to 35% of patients in JULIET and approximately half of patients in ZUMA-1 ultimately relapsed (7).

**Figure 1:**
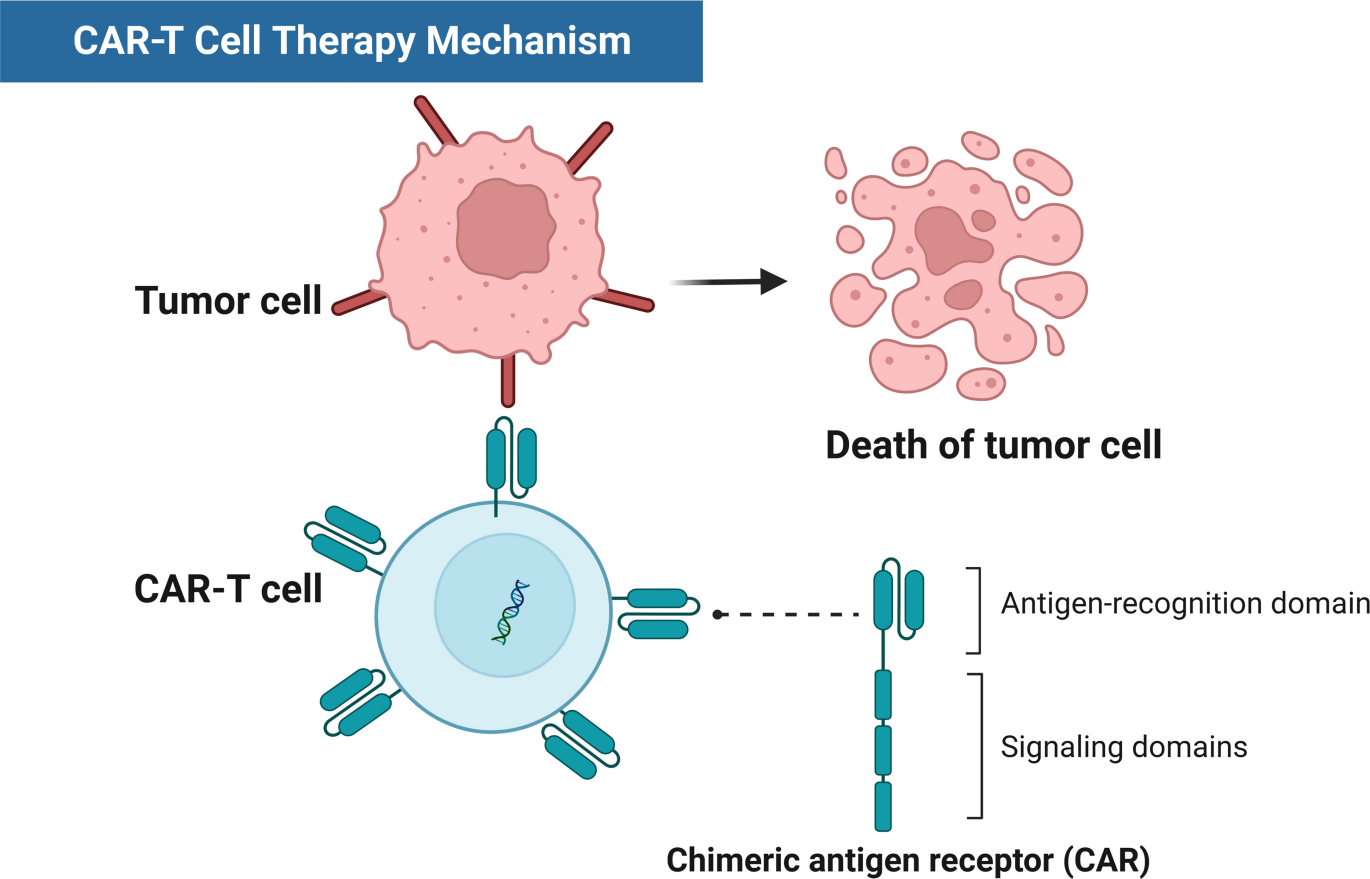
Schematic diagram of CAR-T therapy mechanisms.

With the widespread application of CAR-T therapy, an increasing number of patients have been successfully treated; at the same time, growing attention has been drawn to resistance to this therapy. Numerous studies have tried to define some factors that are associated with the responses and outcomes following CAR-T therapy, especially in lymphoma patients. Taking advantage of these factors, we can predict the responses to CAR-T immunotherapy and further recognize those who may benefit most from the therapy. In addition, for patients manifesting the characteristics of a high risk of resistance or relapse, the introduction of consolidation or maintenance treatment following CAR-T therapy could be considered in certain clinical circumstances. Furthermore, to address the failure of CAR-T therapy, it is necessary to know the corresponding mechanisms. In this article, we review the biomarkers related to short/long-term efficacy in R/R lymphomas of B-cell origin and discuss the mechanisms of resistance to CAR-T therapy (**Table 1** and **Figure 1**).

**Table 1:** Summary of mechanisms responsible for CAR-T resistance/recurrence and possible solutions.

Summary of Mechanisms responsible for CAR-T Resistance/Recurrence		Possible Solutions
**Antigen Positive Relapse**	**CAR-T Cell Costimulatory Domain** (5, 8, 9) CD28 or 4-1BB **Source of single-chain variable fragment (scFv)** (10, 11) mouse-derived or human-derived **Age of Patients** (12–17) **T Cell Exhaustion** (18–20)	Incorporating 4-1BB costimulatory domain in designing CARs Incorporating human-derived scFv in designing CARs Considering universal CAR-T in the elderly Combining CAR-T therapy with immune checkpoint blockade
**Antigen Negative Relapse**	**Antigen epitope alteration** (11, 12, 21–26) CD19 gene mutation Alternative splicing **Defects in CD19 Processing** (27, 28) Loss of CD81 **Epitope Concealment** (27, 29) CAR gene unintentionally introduced into tumor cells **Immune Pressure** (12, 30, 31) CD19-negative tumor proliferating **Pedigree Transformation** (12, 27, 32–37) Tumor dedifferentiation Cytokine induced myeloid differentiation **The Increase of Macrophages Leads to the Loss of Reversible Antigen** (12, 38–40)	Dual/multi-targeted CAR-T; sequential infusion of CAR-T cells targeting different antigens Dual/multi-targeted CAR-T; sequential infusion of CAR-T cells targeting different antigens Optimize the production process Dual/multi-targeted CAR-T; sequential infusion of CAR-T cells targeting different antigens Dual/multi-targeted CAR-T; sequential infusion of CAR-T cells targeting different antigens Positive control of CRS Dual/multi-targeted CAR-T; sequential infusion of CAR-T cells targeting different antigens
**Other Mechanisms**	**Expression of inhibitory ligands** (27, 41–48) **Resistance to the immune system** (19, 49–52)	Combining CAR-T therapy with immune checkpoint blockade Combining CAR-T therapy with proapoptotic agents

## Biomarkers for therapeutic response

### Patients’ baseline characteristics

A series of studies indicated some baseline characteristics of patients, such as age, performance status, disease stage, and levels of cytokines, as well as some biochemical indicators that may result in poor response to CAR-T immunotherapy. In a retrospective cohort evaluating axi-cel in real-life clinical practice at multiple centers, Loretta and colleagues analyzed the relationship between patients’ baseline characteristics and the response after therapy (53). The results showed that patients with older age (≥60 years old), better performance status (ECOG 0~1), nonbulky disease, prior high-dose therapy/autologous stem-cell transplantation (HDT/ASCT) or normal lactate dehydrogenase (LDH) had a higher 12-month CR rate (53), and multivariable analysis revealed that the best response of a CR at 12 months was associated with older age and normal LDH at the time of conditioning (53). Other studies confirmed the negative role of elevated LDH levels in response to CAR-T therapy (54, 55), which implicated high lymphoma burdens and aggressive disease courses (56). The disease stage of lymphoma could also reflect the response, as stage IV was a premonitory factor for 1-year progressive disease with an odds ratio of 9.335 (57). Since LDH and disease stage could both predict response after CAR-T therapy, the age-adjusted International Prognosis Index (aaIPI), including the above two factors, was reported to correlate significantly with 6-month complete metabolic response (CMR) after CAR-T therapy (58). It seemed that among the baseline characteristics, factors involved with tumor burden, such as LDH, had the most predictive value, and coincidentally, high total metabolic tumor volume (TMTV, >80 mL) at infusion was definitely predictive of early resistance within one month following treatment, with a hazard ratio of 4.35 (59).

Furthermore, tumor-related factors such as TP53 alterations that were routinely analyzed in DLBCL strongly affected the effectiveness. Roni et al. (60) conducted an observational study to determine the predictive role of TP53 abnormities in CD19-directed CAR-T therapy. They found that several disease-related features comprising TP53 alterations, primary refractory disease and stable disease (SD) or progressive disease (PD) prior to CAR-T-cell administration were connected to a lower likelihood of achieving a CR (60). Among these features, TP53 alterations remained an independent predictor of response in a multivariable model, with CR rates by Day 90 of 65% versus 34% in favor of wild-type TP53 (60). The predictive values of clinical factors were evaluated, and pretreatment increases in IL-6 showed a significant association with a lower objective response rate and fewer opportunities to achieve a durable response (61).

### CAR-T-cell functional features

CAR-T-cell functions play a crucial role in achieving and maintaining disease remission, among which CAR-T-cell expansion is thought to represent a key parameter of treatment response. First, the association of active CAR-T-cell expansion with response was found in the ZUMA-1 trial (4), as the area under the curve of CAR-T-cell levels in responders was 5.4 times as high as the value in nonresponders (4). Several other studies confirmed this kind of association (10, 58, 62, 63). In a representative study, patients were divided into weak expanders and strong expanders according to the peak blood concentrations of CAR-T cells (CAR-T-Cmax) (62). The objective response (CR+PR) rates at 30 days were 91% vs. 40% (P = 0.02) in favor of strong expanders, with only one not responding among eleven strong expanders (62). In comparison with patients who did not respond, responders (CR+PR) had significantly higher CAR-T-Cmax levels (median 22.06/mL vs. 3.02/mL, P=0.006) (62).

Based on the cell surface phenotype after antigen stimulation, T cells exist as naïve (T_N_), memory (T_M_) and effector (T_E_) subsets (64), and the T_M_ subset is additionally partible into central memory (T_CM_) and effector memory (T_EM_) subpopulations (64–68). Memory stem T lymphocytes (T_SCMs_), a subtype of memory T cells (67), possess a distinct transcriptional profile and function in other T-cell subsets (69–71). In contrast to T_N_ cells, T_SCMs_ show high expression of CD95, CXCR3, CD58, and IL2Rb (69–71). Preliminary studies aimed to uncover the association of the subsets of CAR-T cells with the expansion and subsequent duration of CAR-T cells *in vivo* (72). The results showed that in 14 B-cell lymphoma patients, a high proportion of a subgroup of T cells possessed cell-surface CD8, CD45RA and CCR7, which is most in accordance with the phenotype of T_SCMs_ and promised better *in vivo* expansion (72). Fraietta et al. (73) further confirmed that in advanced, previously heavily treated CLL patients receiving CAR-T therapy, CAR-T cells from subjects who achieved a complete response exhibited an upregulation of genes involved in the memory phenotype, while the counterparts from nonresponders were enriched in exhaustion- and apoptosis-related genes (10). Similarly, the frequencies of CD8+ T cells with memory-related attributes in large B-cell lymphoma (LBCL) patients who achieved a CR at 3 months were threefold higher than those in patients who achieved a PR/PD (74). A recent analysis of ZUMA-1 patients also demonstrated this correlation, indicating that greater proportions of the T-cell subset expressing CCR7 and CD45RA in the apheresis product and the final infusion product were consistently negatively associated with product doubling time (DT) (61). The median DT in nonresponders (2.1 days) was significantly longer than that in responders (1.6 days) (P =0.0067), and a longer DT exactly predicted a lower response rate (61). In contrast, Sylvain et al. (58) showed different results: higher frequencies of CAR-T cells that showed an EM-like phenotype and decreased percentages with naïve properties were predictive of enhanced efficacy (58). Thus, in future studies, the issue of whether the *in vitro* conditions of T-cell growth in different studies, including the sorting methods of T cells, culture protocols and *in vitro* culture time, may impact the final product composition should be addressed.

T-cell exhaustion has already become a noticeably negative prognostic factor for response to genetically modified T-cell therapy. Exhausted T cells express high levels of inhibitory molecules such as PD-1, TIM-3 and LAG-3, which are so-called immune checkpoints (18). Olivia and colleagues elaborated on the phenomenon that greater proportions of CAR-T cells that possess the cell-surface inhibitory molecules mentioned above at the initial stage of tumor eradication led to deficiencies in CAR-T-cell function attributes such as expansion and persistence and subsequently a lower likelihood of tumor eradication responses (18). Increased percentages of CD8+ T cells with expression of PD-1 and concurrent LAG-3 as well as CD4+ T cells with expression of PD-1 in apheresis products were demonstrated to be associated with failure to respond (18). The results also illustrated that greater CD8+ LAG-3+ T-cell numbers and concurrent deficiencies in producing cytokines such as TNF-α resulted in a high risk of early therapeutic failure (18). Similarly, another study demonstrated that significantly decreased frequencies of preinfusion CD8+PD-1+ CAR-T cells were seen in CR subjects in comparison with those who achieved PR or failed to respond (73).

### Other biomarkers

A highly immunosuppressive milieu exhibited a negative impact on the functional properties of CAR-T cells and showed an association with limited responses. As described by Gunilla and colleagues, the best sign of a response was proven to be good immune attributes before CAR-T-cell infusion with high plasma levels of myeloid activation markers such as IL-12 and DC-lamp or lymphocyte effector markers such as Fas ligand and TRAIL (75). Moreover, responders possessed low levels of IL-6, IL-8, NAP3, sPD-L1 and sPD-L2 and fewer monocytic myeloid-derived suppressor cells, which featured the expression of CD14 and CD33 without the expression of HLA-DR (75).

## Biomarkers for long-term efficacy

Poor responses always indicate poor outcomes, and the previously described patients’ baseline characteristics, including performance status, aaIPI and LDH, were all predictive factors for long-term efficacy. However, only increased levels of LDH prior to CAR-T-cell infusion were prognostic for inferior PFS and OS in multivariate analysis following tisa-cel therapy in the JULIET trial (76, 77). The results from a large real-world retrospective study in which axi-cel was administered as standard treatment to 275 R/R LBCL patients showed that poor performance status (ECOG 2-4) and high LDH levels were related to shorter PFS and OS (53, 77). In addition, another real-world study recognized elevated LDH and two or more extranodal sites at the time of decision to receive CAR-T treatment and elevated CRP, two or more extranodal sites, and TMTV exceeding 80 mL at the time of treatment as negative predictive factors for PFS and OS (59). IPI and aaIPI, which utilize patients’ baseline characteristics to forecast outcomes of DLBCL, were also found to be prognostic (77). According to the findings by Garcia-Recio and colleagues, high-risk aaIPI (≥2) indicated worse OS, while both high-risk IPI (≥3) and aaIPI predicted shorter PFS (78). Sylvain and colleagues revealed similar results that high-risk aaIPI indicated inferior PFS and OS (58). In addition to IPI, Gray and colleagues found that CRP ≥ 11 was a risk factor for survival at 1 year (P=0.019), while absolute lymphocyte count ≥ 0.50 at collection (P=0.043) and tocilizumab exposure (P=0.005) were protective factors (57). The findings of Arushi et al. (79) indicated that the optimal time when CAR-T cells would be incorporated also counts. To determine whether the previous intensity of treatment would influence the outcome, patients who could undergo CAR-T therapy at the earliest possible indication, which was either after two lines of chemotherapy or after ASCT following two lines of chemotherapy, were identified as CAR-T[early]; otherwise, they were identified as CAR-T[late] (79). At the 1-year follow-up, the EFS rates in the CAR-T[early] group and CAR-T[late] group were 48% and 30%, respectively, with marginal significance (P= 0.055), and similarly, the OS rates were 75% vs. 56% in favor of the CAR-T[early] group (P = 0.053) (79).

The impacts of tumor intrinsic factors on outcomes after CAR-T therapy were also determined. Hill and colleagues (80) performed whole exome and transcriptome sequencing in 121 R/R DLBCL patients and divided these patients into several subtypes according to their genetic features. The patients were indicated to be BN2, A53, EZB, MCD, N1, or ST2 subtypes or unclassifiable (UC) on basis of the criterion reported by Wright et al. (81) and to be C0, C1, C2, C3, C4 or C5 subtypes as described by Chapuy et al. (82). Patients with the C5/MCD subtype and C2/A53 subtype were found to have better outcomes (80). Patients with the C3/EZB subtype had worse PFS, as well as those whose sequencing results revealed mutations in specific genes, including BCL-2 and MYC (80). As described above, TP53 alterations lead to inferior responsiveness, and DLBCL patients with tumors harboring TP53 alterations had inferior outcomes following CD19-directed CAR-T immunotherapy, especially in subjects who received genetically modified T cells with a second-generation CAR comprising a 4-1BB costimulation domain (60). Leveraging the high resolution of whole genome sequencing (WGS), Michael and colleagues revealed that chromothripsis and APOBEC, which reflect genomic complexity, as well as certain genomic abnormities involving RHOA and RB1 may explain the treatment failure in aggressive B-cell lymphoma patients, with 93.8% of those who relapsed having at least one of the genomic abnormities mentioned above (83).

In a multicenter retrospective analysis, Andrea et al. (84) assessed early PET-CT response according to the Deauville five-point scale in R/R LBCL patients as a predictive factor. They found that patients who achieved early responses of Deauville score (DS) of 1 to 2 exhibited remarkable long-term survival, and in further multivariable analysis, only DS groups showed significance of prediction to relapse following axi-cel or tisa-cel (84). The PFS rates at 12 months were 77.1%, 63.5%, 43.5%, and 0% in the DS 1-2, DS3, DS4 and DS5 groups, respectively, and the OS rates were 87.1%, 86.2%, 61.7%, and 38.1%, respectively (84). Circulating tumor DNA (ctDNA) has become a marker for risk stratification and a predictor of the efficacy of chemotherapy in DLBCL patients (85–88), and preliminary data indicated that molecular remission determined based on ctDNA monitoring successfully predicted the outcomes (74, 88). Further study conducted by Matthew and colleagues frequently monitored ctDNA in LBCL patients treated with Axi-cel from the initiation of the lymphodepleting process to 1 year following CAR-T infusion or disease progression (88). Compared with patients without detectable ctDNA by next-generation sequencing at 28 days after infusion, in whom neither PFS or OS were reached, those with detectable ctDNA had significantly shorter median PFS (3 months) and OS (19 months) (88). In addition, 70% (23/33) of the patients with durable remission had undetectable ctDNA at 1 week; in contrast, the proportion in those with progressed disease was as low as 13% (4/31) (P<0.0001) (88). In patients who achieved PR or SD at Day 28 after axi-cel infusion, among 17 patients with simultaneous detectable ctDNA, 15 patients finally relapsed, while among 10 with simultaneous undetectable ctDNA, only 1 relapsed (P<0.0001) (88), which validated the predictive value of ctDNA assessment after CAR-T therapy.

Apart from being associated with the therapeutic response, transgene copies of CAR-DNA, which indicate that CAR-T cells continuously grow and exist, are related to the long-term response. As reported by Francis et al. (62), patients were divided into weak expanders and strong expanders according to CAR-T-Cmax. Nine of eleven strong expanders were alive, with 8 achieving durable remission (62). In contrast, among weak expanders, except for 2 requiring additional treatment, 8 out of 10 had progressed lymphoma and eventually died (62). At a median follow-up of 121 days, the 1-year PFS rates were 71% and 0%, respectively (P<0.001), in favor of strong expanders (62). Features of CAR-T-cell biology also have the potential to predict long-term efficacy, with sustained remission related to a greater proportion of T cells with the memory-like phenotype of CD8+CD27+CD45RO- prior CAR-T-cell production (73). In contrast, Zinaida and colleagues identified a group of T cells expressing CD4 and Helios, and with single-cell proteomic profiling, these cells were found to be nonclonal and to possess the characteristics of T regulatory (T_Reg_) cells (32). Furthermore, a link between increased CAR-T_Reg_ cells at 7 days after infusion and clinical progression was observed (32).

With insight into the factors that may influence CAR-T-cell function, a novel population quantitative systems pharmacology (QSP) model was designed to forecast the response to CAR-T therapy (89). Anna and colleagues screened more than two thousand factors related to cytokines, CAR-T-cell phenotype features, and metabolic tumor measurements and subsequently proposed a predictive clinical composite score (CCS) (89). They found a cutoff CCS_TN_ value of 0.00136, and survival was totally different between subjects with a CCS_TN_ value above and below the cutoff (89). The median PFS was 11 months and 2 months, respectively, in favor of the subjects with CCS_TN_ values exceeding 0.00136 (P = 0.014) (89). The median OS in subjects who had CCS_TN_ values that surpassed the cutoff was not reached and was significantly longer than the median OS of 2 months in the counterparts that had CCS_TN_ values lower than the cutoff (P = 0.003) (89).

Interestingly, an association between alterations in the intestinal microbiome and survival was observed. A higher abundance of *Faecalibacterium* and members of the genus *Ruminococcus* in the intestinal microbiome was found to be associated with increased monocytes, neutrophils and lymphocytes (90). The metabolites produced by many bacteria in the intestinal microbiome, such as butyrate, can regulate the differentiation of regulatory T (Treg) cells, induce the expression of the transcription factor T-bet and mediate IFN-γ-producing Treg cells or conventional T cells (91, 92). Reported findings from a retrospective cohort including 228 R/R B-cell malignancy patients showed that antibiotic administration within 4 weeks prior to CAR-T-cell infusion, especially piperacillin/tazobactam, imipenem/cilastatin and meropenem (PIM), which may alter the specific intestinal microbiome, was significantly related to inferior PFS and OS (93).

## CAR-T treatment resistance/recurrence mechanism

### Antigen positive relapse

#### CAR-T-cell costimulatory domain

Costimulatory domains may influence the stability of CAR-T-cell therapy (5, 8, 12). A preclinical study by Zhao et al. (9) showed that the 4-1BB costimulatory domain was more persistent than the CD28 costimulatory domain. In this study, they tested the persistence and function of different CAR-T cells containing CD28 or 4-1BB costimulatory domains (9). CAR-T cells with 4-1BB costimulatory domains could induce the expression of IRF7 and IFNB1 (9), which can improve the antitumor effect of T cells. The other two studies also found the superior functionality of CD19 CAR-T cells with the 4-1BB costimulatory domain over those with the CD28 costimulatory domain (94, 95).

#### Source of single-chain variable fragment

The sources of the single-chain variable fragment (scFv) mainly include mouse-derived and human-derived fragments. CARs incorporating human-derived scFv could lessen their antigenicity, thus raising the durability of CAR-T cells (11). However, most anti-CD19 CARs used in clinical trials contain murine scFv, most of which was FMC-63-derived. It was found that binding of CARs containing mouse-origin scFv may trigger human leukocyte antigen-restricted T-cell-mediated immunomodulatory responses (10). This process can lead to a sustained reduction in CAR-T-cell persistence, which can lead to early relapse.

#### Patient age affects the quality of CAR-T cells

Kotani et al. (13) found that CAR-T cells from mice of older age had a short lifespan and poor capacity for expansion *in vivo*, although they had good cytotoxicity *in vitro*, whereas CAR-T cells from mice of younger age showed more active cell proliferation and distinction than those from aged mice. This suggests that the different results may be related to the age-dependent phenotype of CAR-T cells. Guha et al. (14) examined CAR-T cells from young and old donors. They found that the transduction of T cells by CAR-T cells from old donors was significantly less efficient than that of CAR-T cells from young donors. Moreover, CAR-T-cell function was impaired. Thus, older CAR-T cells can induce CD19-positive relapse, mainly due to poorer persistence and efficacy, resulting in longer average event-free survival in pediatric patients and young adults than in adults after CAR-T-cell treatment (12, 15–17).

#### T-cell exhaustion

As described above, inhibitory receptor phenotype and expression are associated with clinical response and long-term efficacy. High expression of immune checkpoint molecules indicative of T-cell exhaustion, such as PD-1, TIM3, and LAG3, can destabilize immune synapses and suppress functional immune responses (19, 20), leading to resistance or relapse after CAR-T-cell treatment (12, 18).

### Antigen negative relapse

To date, antigen loss has been the most frequently studied mechanism of relapse or resistance in CAR-T cells after treatment (27, 96–99).

#### Antigen epitope alteration

Recent studies have shown that the CD19 gene contains exons 1-13, in which exon 4 specifically encodes the FMC63 binding sites in the CD19 CAR (11, 12). Orlando et al. (21) examined flow cytometry results in 17 patients and showed that 12 patients were CD19 negative. All samples from CD19-negative patients underwent RNA and/or DNA sequencing (22). They found CD19 mutations in all 12 samples from patients who relapsed. These CD19 mutations occurred in exons 2-5, and each patient had a unique insertion or deletion in exons 2-5. The study also reported that 8 patients had a loss of CD19 heterozygosity during relapse. In addition, mutations in the CD19 gene have also been reported in refractory DLBCL (23). Alternative splicing is one of the mechanisms that leads to antigen epitope alteration of CD19, which leads to tumors escaping CAR-T treatment. Other tumors also have the same mechanisms, such as trastuzumab resistance due to the splicing of exon 16 of the HER2 gene in breast cancer tissue and vemurafenib resistance due to the splicing of BRAF (V600E) in melanoma tissue (12). Sotillo et al. (24) analyzed CD19-positive samples from the same patient before CAR-T-cell treatment and CD19-negative samples at relapse. They found that a mutation in exon 2 of the CD19 gene in the patient’s tumor cell samples led to the loss of CD19. The inhibition of the SRSF3 gene resulted in an increase in CD19 exon 2 skipping, and lower levels of SRSF3 were found in patients who relapsed, suggesting that the deletion of the SRSF3 gene is associated with CD19 mutations. Although the CD19 mutant retained its function and prevented cell proliferation and B-cell receptor (BCR) signaling defects (25), it failed to trigger CD19-targeted CAR-T-cell killing, leading to tumor escape (12). Jacoby et al. (26) investigated changes in pedigree markers in mice after CAR-T treatment. Using CD19 exon-specific primers, they detected a loss of the transcription of splicing exons 1-3 in E2A-PBX cell lines from CD19-negative mice. This suggests that a loss of exon 2 leads to negative expression of CD19, causing disease relapse (26).

#### Defects in Ag processing

Defective CD19 processing is a currently reported cause of resistance to blinatumomab (28, 100), and this mechanism has been linked to CD81. CD81 is a protein that regulates the maturation and transport of the CD19 protein from the Golgi apparatus to the cell surface as a chaperone. Therefore, the deletion of CD81 prevents the processing and maturation of CD19 in the Golgi matrix (27). In one patient, transcriptional downregulation led to loss of CD81, resulting in a negative relapse after blinatumomab treatment. This reported mechanism of resistance to blinatumomab may also occur with CAR-T-cell therapy, although this mechanism has not yet been reported (27).

#### Epitope concealment

During CAR-T-cell production, the CAR gene can accidentally enter tumor cells, and its product binds to the CD19 epitope on the surface of tumor cells, thereby masking its recognition and resistance to CTL019, an FMC63-derived CAR-T product (29). Ruella et al. (29) found CAR transplantation-induced disease relapse in one patient after CTL019 therapy, and they did not detect CD19 tumor cells in the patient by flow cytometry. After further analysis, they concluded that CAR19 bound to CD19 on the surface of leukemia cells, resulting in an epitope that could not be detected by flow cytometry; therefore, CAR-T cells could not recognize tumor cells.

#### Immune pressure

By killing targeted tumor cells, nontarget tumor cells clone in large numbers and cause relapse (12). Grupp et al. (30) identified a small number of CD19-negative tumor cells derived from clones that were present in a patient with CD19-negative relapse after CAR-T-cell treatment. This suggests that these CD19 antigen-negative tumor cells proliferate under selective CD19 CAR-T therapeutic pressure, leading to CD19-negative relapse (12). Fischer et al. (31) analyzed bone marrow and peripheral blood specimens from untreated CD19-positive patients and healthy subjects. They found weak expression of both the full and partial deletion isoforms of CD19 exon 2 in samples from CD19-positive patients, and similar results were obtained in samples from healthy subjects. These results suggest that some B cells with a loss of CD19 expression may have existed before CAR-T therapy, but after CAR-T therapy targeted killing of CD19-positive cells, CD19-negative cell clones proliferated, resulting in CD19-negative recurrence.

#### Pedigree transformation

Pedigree conversion can lead to antigen disappearance and may result in a broader phenotypic change (27, 101). Gardner et al. (33) found that two patients with recurrent disease lost expression of gonadal lineage B antigens, including CD19, and gained expression of myeloid antigens. They investigated two mechanisms of gene switching. The first mechanism was the occurrence of IgH reprogramming in recurrent myeloid stem cells and the reprogramming or dedifferentiation of earlier B lymphoblastoid stem cells. While flow cytometry did not show a spectral transition early in CAR-T treatment, this transition appeared later, suggesting that CAR-T-cell therapy provides a selective advantage of spectral transition (33). Cytokine levels during CRS may also lead to genealogical transitions. Two patients that underwent genealogical transition suffered more severe CRS than those without genealogical transition (33). CRS severity has been shown to be strongly correlated with IL-6 levels (12, 34, 35), and IL-6 is a key factor in myeloid differentiation (33, 36, 37). Cohen et al. (36) found that IL-6 was able to induce the production of the early myeloid marker CD33 on B1 cells. They also found that IL-6 was able to induce CD45 antigen production and that the CD45 gene product was able to regulate growth, including some hematopoietic factors (36). Moreover, the promotion of myeloid differentiation by IL-6 may also be associated with the induction of specific chromosomal translocations. Tocilizumab, an anti-human IL-6R antibody that was already proven to be effective in alleviating severe CRS, may also be able to prevent IL-6-induced myeloid differentiation.

#### The Increase in macrophages leads to the loss of reversible antigen

Macrophagocytosis is a phenomenon in which lymphocytes can release surface molecules from antigen-presenting cells, which they bind *via* “immune synapses”, which involves the transfer of plasma membrane fragments from the presenting cells to the lymphocytes (38). This is an active transfer triggered by antigen receptor signals (39). Hamieh et al. (40) used a mouse model to simulate the reuptake of CAR-T cells after infusion. They labeled all cells with CD19 fluorescence and cultured them with CAR-T (19-BB-ζ) cells. They found that CD19 expression was increased in a large proportion of CAR-T cells, while it was decreased in tumor cells. The transfer of CD19 protein from tumor cells to T lymphocytes—so called trogocytosis, could decrease target density on tumor cells and abate T cell activity by promoting fratricide T cell killing and T cell exhaustion (35). They also found that mice with the CD19 gene knocked out had a weaker response to low doses of CAR-T cells (40). This suggests that a reduction in target antigen density may lead to CAR-T-cell resistance, resulting in disease relapse.

### Other recurrence mechanisms

#### Expression of inhibitory ligands

The programmed death-1 (PD-1)/programmed death ligand-1 (PD-L1) axis is a pathway that inhibits immune checkpoints. PD-L1 is known to be expressed in lymphomas (102). The binding of these ligands to their receptors inhibits the functions of T cells and limits tumor cell killing, thereby allowing immune escape (27, 41–43). It is now possible to combine PD-L1 inhibitors with CAR-T cells to enhance the effectiveness of CAR-T therapy. Song et al. (44) considered the combined use of CAR-T-cell therapy and PD-L1 antagonists. They found that there is indeed a synergistic effect between CAR-T cells and PD-1 antagonists in the treatment of malignant diseases (45). They also concluded that the disruption of the PD-1 pathway can restore efficient functioning of CAR-T cells, suggesting that PD-1 blockade may be an effective strategy to improve the efficacy of CAR-T-cell therapy (44). Rafiq and colleagues developed CAR-T cells capable of secreting anti-PD-L1 antibodies (46, 47). These cells may be effective in enhancing the efficacy of CAR-T therapy in a mouse model (46, 47). The above data suggest that PD-L1 is a factor influencing CAR-T therapy. Nanobody-based CAR-T cells have been shown to have higher affinity and are easier to produce than single-chain antibody-based CAR-T cells (48). Xie et al. (103, 104) found that nanobodies targeting PD-L1 together with CAR-T cells slowed tumor growth and improved CAR-T-cell function. This suggests that nanobody-based PD-L1 inhibitors could play an important role in the treatment of blood diseases in the future.

#### Resistance to the immune system

Recently, increasing data have shown that the mechanism of tumor cell apoptosis is impaired, which may cause tumor cells to resist immune killing by CAR-T cells (27). A study by Singh et al. (49) found that defects in death receptor signaling pathways in lymphomas lead to resistance to CART19 and consequently reduced CAR T-cell function. Their studies showed that the deletion of genes related to the proapoptotic death receptor signaling pathway causes the resistance of CAR-T cells to killing (49), leading to disease relapse. Their study also found that CR patients had higher death receptor signals than PR patients. Dufva et al. (50) found that death receptor signaling is an important mediator of CAR T-cell toxicity and reactivity. These receptors can enhance cancer immunotherapy. In addition, genes involved in the death receptor pathway can promote the efficacy of CAR-T-cell therapy and exert more extensive tumor killing (51). Although the extent of this mechanism in hematological malignancies is still unclear (27), it could be used as a tool to improve the efficacy of CAR-T therapy (52).

## Conclusion

To conclude, great efforts have been invested in the identification of biomarkers to predict efficacy and outcomes, and as a consequence, we could recognize patients who have greater opportunities to respond and further achieve long-term survival from CAR-T therapy. On the other hand, for patients who respond to CAR-T therapy, these biomarkers facilitate the identification of those who have a high risk of relapse, which warrants the development of preemptive strategies to prolong the response. As outlined in this review, various factors, including resistant tumor cells, dysfunctional CAR-T cells and a hostile tumor microenvironment, could lead to CAR-T therapy failure. Dealing with resistance and relapse after CAR-T therapy is still difficult. Based on different mechanisms responsible for resistance, many novel therapeutics, such as CAR-T therapy directed at new targets, immune checkpoint inhibitors, immunomodulatory agents, bispecific antibodies, and drug-conjugated antibodies, are under investigation and provide new hope to patients in the post-CAR-T era.

## Author contributions

HX and NL searched the literature and drafted the manuscript, and GW and YC designed the article structure and revised the manuscript. All authors contributed to the article and approved the submitted version.
